# Further Characterization of Functional Domains of PerA, Role of Amino and Carboxy Terminal Domains in DNA Binding

**DOI:** 10.1371/journal.pone.0056977

**Published:** 2013-02-25

**Authors:** J. Antonio Ibarra, Claudia M. García-Zacarias, Cristina Lara-Ochoa, Alejandro Carabarin-Lima, J. Sergio Tecpanecatl-Xihuitl, Ernesto Perez-Rueda, Ygnacio Martínez-Laguna, José L. Puente

**Affiliations:** 1 Departamento de Microbiología Molecular, Instituto de Biotecnología, Universidad Nacional Autónoma de México, Cuernavaca, Morelos, México; 2 Centro de Investigaciones en Ciencias Microbiológicas, Benemérita Universidad Autónoma de Puebla, Puebla, México; 3 Departamento de Ingeniería Celular y Biocatálisis, Instituto de Biotecnología, Universidad Nacional Autónoma de México, Cuernavaca, Morelos, México; University of Kentucky College of Medicine, United States of America

## Abstract

PerA is a key regulator of virulence genes in enteropathogenic *E. coli*. PerA is a member of the AraC/XylS family of transcriptional regulators that directly regulates the expression of the *bfp* and *per* operons in response to different environmental cues. Here, we characterized mutants in both the amino (NTD) and carboxy (CTD) terminal domains of PerA that affect its ability to activate the expression of the *bfp* and *per* promoters. Mutants at residues predicted to be important for DNA binding within the CTD had a significant defect in their ability to bind to the regulatory regions of the *bfp* and *per* operons and, consequently, in transcriptional activation. Notably, mutants in specific NTD residues were also impaired to bind to DNA suggesting that this domain is involved in structuring the protein for correct DNA recognition. Mutations in residues E116 and D168, located in the vicinity of the putative linker region, significantly affected the activation of the *perA* promoter, without affecting PerA binding to the *per* or *bfp* regulatory sequences. Overall these results provide additional evidence of the importance of the N-terminal domain in PerA activity and suggest that the activation of these promoters involves differential interactions with the transcriptional machinery. This study further contributes to the characterization of the functional domains of PerA by identifying critical residues involved in DNA binding, differential promoter activation and, potentially, in the possible response to environmental cues.

## Introduction

Enteropathogenic *Escherichia coli* (EPEC) is a common cause of acute diarrhea mainly in infants living in developing countries [1], [2]. EPEC interaction with intestinal epithelial cells differs from that observed in other bacteria like *Salmonella* or *Shigella*, as EPEC is not an invasive pathogen. This interaction involves a complex series of events that for research purposes has been divided in three stages. The first stage is the colonization process during which localized adherence occurs, a phenotype considered to be a common property of the typical EPEC [1], [3], [4]. In the second stage a cohort of bacterial effectors is translocated into the host cell cytoplasm using a type III secretion system (T3SS) encoded by the Locus of Enterocyte Effacement (LEE) [5]. Translocated effectors include Tir (Translocated intimin receptor), which upon its translocation is inserted into the host cell membrane. The third phase is characterized by the intimate association of the bacteria with the host cell membrane, which is mediated by the interaction of the outer membrane protein intimin with Tir. These events lead to the generation of the attaching and effacing (A/E) lesion, characterized by the dissolution of the intestinal brush border and loss of epithelial microvilli (effacement) at the sites of bacterial attachment and by the formation of actin-rich pedestal-like structures underneath the adherent bacteria [6].

Localized adherence is associated with the production of a type 4 fimbriae known as the bundle-forming pilus (BFP) [7]. BFP has been shown to have an essential role during human infection [8] and as an adherence factor *in vitro* [9]. The proteins responsible for the biogenesis of the BFP filament are encoded by the 14-gene *bfp* operon located in the EPEC adherence factor (EAF) plasmid or pEAF [10], [11]. The BFP structural subunit is encoded by *bfpA*, the first gene of the *bfp* operon [12], [13]. The *bfp* genes are expressed from a promoter located upstream of *bfpA* during the exponential phase of growth at 37°C in tissue culture medium and are negatively modulated by ammonium and by temperatures below or above 37°C [14], [15].

The transcriptional activation of *bfp* requires the product of the *perA* gene, the first gene of the *per* operon localized 18 kb downstream of *bfpA* in pEAF [16], [17]. *perA* encodes a 274 amino acids protein that belongs to the AraC/XylS family of transcriptional regulators and has been shown to be an essential virulence regulator for EPEC in the human host [8]. Frameshift mutations in the *perA* gene of typical EPEC clinical isolates clinical isolates were associated to a reduction in adherence and BFP expression [18], [19].

The closest homologues of PerA (also known as BfpT), such as Rns, CfaR, CsvR, FapR, FasH and AggR, are all positive regulators of fimbrial expression in different *E. coli* pathovars (reviewed in [20], [21]). PerA controls its own expression by an autoregulatory mechanism that responds to the same signals as those previously described for *bfpA* [22]. Activation of the *per* operon also allows the expression of *perC*, encoding a protein with no evident similarity to other proteins in the data bases, which has been shown to specifically activate the promoter of the *LEE1* operon and thus of *ler*, the gene encoding the master regulator (Ler) of LEE gene expression in A/E pathogens [5], [23], [24], [25]. Altogether, these observations confirm the relevance of PerA in the regulatory network of virulence genes in EPEC.

The AraC/XylS family of bacterial regulators consists of more than 1500 different proteins [21], [26], which regulate a wide range of cellular processes like carbon and nitrogen metabolism, adaptation to environmental changes, stress response, and virulence [20], [21], [26]. Most of these proteins are composed of two structural domains: a conserved DNA binding domain (DBD), usually situated at the C-terminal domain (CTD), that contains two putative α-helix-turn-α-helix (HTH) motifs shown in some members to be involved in DNA binding and in interactions with the RNA polymerase (RNAP); and a non-conserved N-terminal domain (NTD) that is in many cases required for self-dimerization and effector molecule-binding [20], [21], [26]. In addition to be the most variable region amongst the AraC/XylS family members, the NTD is in some cases absent (*i.e.* SoxS and MarA). PerA is composed of both domains, but has been shown to function as a monomer, in contrast to most members of the AraC/XylS family [24], [27]. This protein binds to a 29-bp-long AT-rich consensus motif at the *bfpA* and *perA* regulatory regions, which is located at different distances from the −35 promoter hexamer [27]. PerA is phylogenetically more related to the subfamily of AraC/XylS-like proteins regulating virulence gene expression [20], [21], [26].

In order to further characterize the role that the NTD and CTD domains play in PerA function, here we introduced different point mutations and deletions in both domains and studied their effect in promoter activation *in vivo* and in DNA binding. As predicted, mutations at the HTH1 and HTH2 motifs caused a significant negative effect in PerA function and DNA binding. Interestingly, specific mutations at the NTD also had a detrimental effect on PerA activity, which in most cases also correlated with a defect in DNA binding. Moreover, mutations at residues near the linker region affected differentially the activation of the PerA-dependent promoters or generated variants that were less responsive to the presence of ammonium in the growth medium. Our results further sustain the importance of both the CTD and NTD domains of PerA on promoter activation by demonstrating their role in DNA recognition. Our results also suggest additional roles for these domains such as their putative involvement in the response to environmental signals and promoter activation, but also suggest that within these domains PerA contains a sequence motif that may be involved in the response to environmental signals and differential promoter activation.

## Materials and Methods

### Bacterial Strains and Growth Conditions

The strains and plasmids used in this study are listed in Table 1. Luria-Bertani (LB) broth [28] or Dulbecco’s modified Eagle’s medium (DMEM) without sodium pyruvate, containing 0.45% (w/v) glucose and L-glutamine (584 mg/l) (Gibco BRL Life Technologies) and supplemented with 1% LB (v/v), were used for growth of cultures at 37°C. When indicated the medium was supplemented with 20 µM ammonium sulfate (Sigma). When necessary, 100 µg/ml ampicillin or 25 µg/ml kanamycin (Sigma) were added to the media.

**Table 1:** Strains and plasmids used in this study.

Strain or plasmid	Relevant characteristics *	Source or reference
*Strains*		
B171–8	Wild-type EPEC O111:H^−^	[15]
B171-10	EAF plasmid-cured derivative of B171–8	[15]
MC4100	F- *araD139* (*argF-lac*)*U169 rpsL150 relA1 flbB5301 deoC1 ptsF25 rbcR*	Laboratory strain
BL21/pLysS	Δ*ompT* (*lon*) *hsdS_B_* (*r*_B_ *m_B_*) *gal dcm* (Δ DE3) pLysS (Cm^R^)	Invitrogen
*Plasmids*		
pCS-T	pACYC184 derivative carrying *perA* (*bfpT*)	[22]
pCS-T138A	pCS-T derivative with a I138A change that inserts a HindIII restriction site. SDM	This work
pCST_K14A_	pCS-T derivative carrying PerA_K14A_. SDM	This work
pCST_Y26A_	pCS-T derivative carrying PerA_Y26A_. SDM.	This work
pK3-mut-PerA_Y29A_	pCS-T derivative containing PerA_Y29A_ SDM	This work
pK3-mut-PerA_Q40R_	pCS-T derivative containing PerA_Q40R_ SDM	This work
pK3-mut-PerA_D100A_	pCS-T derivative containing PerA_D100A_ SDM	This work
pK3-mut-PerA_D101A_	pCS-T derivative containing PerA_D101A_ SDM	This work
pK3-mut- PerA_E116A_	pCS-T derivative containing PerA_E116A_ SDM	This work
pCST_S194A_	pCS-T derivative carrying PerA_S194A_. SDM.	This work
pCST_R199G_	pCST derivative carrying PerA_R199G_. SDM.	This work
pCST_K249M-Y255A_	pCS-T138A derivative carrying PerA_K249M-Y255A_. OE-PCR	This work
pCST_Y255A_	pCS-T138A derivative carrying PerA_Y255A_. OE-PCR	This work
pCST_P259A_	pCS-T138A derivative carrying PerA_ P259A_. OE-PCR	This work
pCST_K260STOP_	pCS-T138A derivative carrying a *perA* with an stop codon in position 259. OE-PCR	This work
pAIT_Δ30–49_	pCS-T derivative carrying a deletion in PerA from amino acid residues 30 to 49. R-PCR	[27]
pAIT_Δ70–102_	pCS-T derivative carrying a deletion in PerA from amino acid residues 70 to 90. R-PCR	This work
pAIT_Δ90–176_	pCS-T derivative carrying a deletion in PerA from amino acid residues 90 to 176. R-PCR	This work
pK3-mut- PerA_D168A_	pCS-T derivative containing PerA_D168A_ SDM	This work
pK3-mut- PerA_D177A_	pCS-T derivative containing PerA_D177A_ SDM	This work
pK3-mut- PerA_E234A_	pCS-T derivative containing PerA_E234A_ SDM	This work
pCAT201	pKK232-8 derivative containing the *bfpA-cat* transcriptional fusion fromnucleotides −201 to +76	[15]
pUST166	pKK232–8 derivative containing the *perA-cat* transcriptional fusion fromnucleotides −155 to +21	[22]
pMalC2xa	Vector for constructing MBP fusions	New England Biolabs
pMALT2	pMalC2xa derivative expressing wild type MBP-PerA	[27]
pMAL-PerA_S194A_	pMalC2xa derivative expressing MBP-PerA_S194A_	This work
pMAL-PerA_R199G_	pMalC2xa derivative expressing MBP-PerA_R199G_	This work
pMAL-PerA_K249M-Y255A_	pMalC2xa derivative expressing MBP-PerA_K249M-Y255A_	This work
pMAL-PerA_P259A_	pMalC2xa derivative expressing MBP-PerA_P259A_	This work
pMAL-PerA_D100A_	pMalC2xa derivative expressing MBP-PerA_D100A_	This work
pMAL-PerA_D101A_	pMalC2xa derivative expressing MBP-PerA_D101A_	This work
pMAL- PerA_E116A_	pMalC2xa derivative expressing MBP-PerA_E116A_	This work
pMAL- PerA_D168A_	pMalC2xa derivative expressing MBP-PerA_D168A_	This work
pMAL- PerA_E234A_	pMalC2xa derivative expressing MBP-PerA_E234A_	This work

* Indicates how the mutant was obtained: SDM, site directed mutagenesis; OE-PCR, overlapping extending PCR; R-PCR, reverse PCR.

### CAT Assay

The CAT assay was performed as described previously [15]. Briefly, bacteria from an overnight culture grown in LB with antibiotics at 37°C were centrifuged and resuspended in phosphate buffered saline (PBS) solution, pH 7.4, to an OD_600_ of 1.0. A 1∶50 dilution of the bacterial suspension was done in 50 ml of fresh LB or DMEM and then incubated in a shaking water bath at 210 r.p.m. (Gyromax 902, Amerex Instruments) at 37°C. Samples were collected every hour in order to determine the OD_600_. At OD_600_ = 1.0 or 1.4, one ml samples were taken to determine chloramphenicol acetyltransferase (CAT) activity. Whole cells pellets were collected by centrifuging (16,000×*g*) and then washed with 1 ml of DL-dithiothreitol (TDTT) buffer (50 mM Tris-HCl, pH 7.8, and 30 mM DTT). The bacterial pellet was resuspended in 500 µl of TDTT buffer and sonicated on ice. Intact cells and debris were eliminated by centrifugation at 16,000×*g* for 20 min, and the supernatants were transferred to clean 1 ml microfuge tubes. For determination of CAT activity, 5 µl of each extract were added in duplicate to a 96-well flat-bottom ELISA plate (Corning), followed by 200 µl of reaction mixture containing 1 mM DNTB (5,5′-dithio-bis[2-nitrobenzoic acid] (Research Organics), 0.1 mM acetyl-CoA (Pharmacia) and 0.1 mM chloramphenicol (Sigma) in 0.1 M Tris-HCl, pH 7.8. Changes in absorbance at 410 nm were recorded every 5 sec for 3 min, using a scanning auto reader and microplate workstation, Ceres 900 C and the KC3 Jr. software (Bio-Tek Instruments) set in the kinetic mode. The activities were obtained by interpolation from a standard curve, generated with purified CAT enzyme (Sigma Chemicals) at concentrations ranging from 0 to 2500 U/µl.

The protein concentration of the cell extracts used in the CAT assay was determined using the BCA protein assay kit (Pierce). Bovine serum albumin (BSA) served as the protein standard. Each given value represents the average activity obtained from at least three independent experiments.

### DNA Manipulations

All DNA manipulations were performed using standard genetic and molecular techniques [28]. Plasmid DNA was purified using the High Pure DNA kit (Roche Scientific Inc.) following the manufacturer’s protocol. Restriction and DNA-modifying enzymes were obtained from Roche, New England Biolabs (NEB) or Invitrogen and used according to the manufacturer’s instructions. The oligonucleotides used for amplification by PCR were synthesized at our institute’s oligonucleotide synthesis facility. Oligonucleotide primer sequences are available upon request. PCR reactions were performed in a 100 µl volume using AmpliTaq polymerase (Applied Biosystems) according to the manufacturer’s instructions. Double-stranded DNA sequencing of the *perA* mutants was done with the dideoxy-chain termination procedure, using a Thermo Sequenase cycle sequencing kit according to the manufacturer’s instructions (Amersham).

Plasmids for the expression of mutant versions of the chimeric protein MBP-PerA fusions were constructed as previously described [27]. Briefly, the *perA* gene (GenBank: AB523702.1) was PCR amplified from plasmid pCS-T using oligonucleotides BFPT-BHI-Fw (5′-GGTTAATGCTTGGATCCAAAAAAG-3′) and TAFB-2862R (5′-TTGTTCTGCAGTTCGAGTGCTC-3′) that introduced BamHI and PstI restriction sites (underlined) respectively. Fragments were cloned in frame at the 3′ end of the maltose binding protein (*malE)* gene in pMALC2xa vector (NEB) using the BamHI and PstI sites. All constructs were sequenced and induced proteins (except for PerA_260STOP_) were detected by Western blot with a specific antibody directed to the last 16 amino acids of PerA (data not shown) [27].

### Mutagenesis of perA

PerA mutagenesis was carried out by one of three different methods: 1) using site-directed mutagenesis (SDM) using a Quick Change kit (Stratagene); 2) by overlap-extended PCR (OE-PCR), as previously described [29]; or 3) by reversed PCR (R-PCR). pCS-T plasmid was used as template (Table 1). Oligonucleotide primer sequences are available upon request.

Quick Change kit for SDM mutagenesis was used according to the manufacturer’s protocol (Stratagene). OE-PCR was done as previously described [29]. Briefly, to facilitate the generation of mutants at the C-terminal domain, plasmid pCS-T138A was generated by designing oligonucleotides with a HindIII site in the sequence corresponding to I138 changing this residue for alanine (I138A). The SacI restriction site in plasmid pCS-T was used and a HindIII restriction site at residue I138 in the putative linker of *perA* was inserted, generating the I138A mutation (plasmid pCS-T138A, Table 1). The activity of the PerA_I138A_ mutant was analyzed in EPEC B171-10 carrying the *bfpA* or *perA* transcriptional fusions and showed no significant difference was observed in the activation of both promoters with respect to the wild type (wt) protein (data not shown). The mutagenic strategy to generate alanine substitutions at the C-terminal domain required four primers and two subsequent rounds of PCR reactions for each mutant. In the first round, two PCR reactions using pCS-T138A as template were performed one with a primer containing the HindIII site and the second primer with the mutation in the selected residue; and the other with the complementing mutated primer and the primer containing the SacI site downstream of the 3′ end of the gene. The products of both reactions were purified and treated with T4 DNA polymerase (NEB) to generate blunt ends. The resulting PCR products containing overlapping regions of 47 bp were mixed for an additional round of 5–7 PCR extending cycles. Finally, the two flanking primers containing the HindIII and the SacI sites were added to this reaction for another 25 PCR cycles to amplify the overlapping product. Products of expected size were extracted from agarose gels with Geneclean kit (BIO 101 System), digested with HindIII and SacI and cloned into previously digested pCS-T138A DNA. This last step reconstituted the full *perA* gene but now containing one of the C-terminal mutations.

R-PCR mutagenesis was done as follows: oligonucleotides were designed to generate in-frame deletions of different codon tracks of the *perA* structural gene and the insertion of a BglII restriction site using pCS-T as template. DNA fragments were gel purified, digested with BglII and ligated. Selected clones were verified by restriction profile and then sequenced. PerA derivatives were detected by Western blot (except for PerA_D101A_ and PerA_260STOP_) were detected by Western blot with a specific antibodies directed to the last 16 amino acids of the protein (Figure S1) [27].

### Expression and Purification of MBP-PerA Proteins

Expression and purification of the MBP-PerA fusions was conducted as described previously [27]. Briefly, *E. coli* strain BL21/pLysS was transformed with pMALT2 or its derivatives (Table 1) and grown in LB medium supplemented with 0.2% (w/v) glucose, 100 µg/ml ampicillin and 30 µg/ml chloramphenicol at 30°C in an agitated water bath. The expression of recombinant protein was induced by addition of 0.3 mM isopropyl-β-D-thiogalactopyranoside (IPTG) (Sigma) when the culture reached an OD_600_ of 0.5 to 0.7. Growth continued for another 3 h at 30°C. Bacteria were collected by centrifugation at 4°C, and the pellet was washed once with ice-cold column buffer (10 mM Tris-HCl pH 7.4, 200 mM NaCl, 1 mM EDTA, 10 mM β-mercaptoethanol) and concentrated 100-fold with the same buffer. Cells were frozen and sonicated in ice by five 30-sec pulses with 30-sec resting cycles. Crude extract was obtained by centrifugation at 14,000×*g* for 30 min at 4°C. MBP-PerA proteins were bound to an amylose resin column equilibrated with column buffer at room temperature and eluted with the same buffer but supplemented with 10 mM maltose (Sigma Chemicals). The concentration of the purified protein was determined by the method of Bradford and analyzed by 10% sodium dodecyl sulfate-polyacrylamide gel electrophoresis (SDS-PAGE).

### EMSAs

Electrophoretic mobility shift assays (EMSAs) were carried out as described before [27]. Briefly, PCR-amplified DNA fragments were incubated in binding buffer [25 mM HEPES pH 7.9, 40 mM KCl, 3 mM MgCl_2_, 1 mM dithiothreitol, 0.1 mM EDTA, 5% (v/v) glycerol] at room temperature for 20 min with different concentrations of MBP-PerA in a final volume of 20 to 30 µl. Samples were loaded onto 0.25X Tris-borate-EDTA (TBE) native 6% acrylamide gels and run at 120 V at room temperature. Positive controls were those used in our previous study [27] and included DNA fragments from the *perA* and *bfpA* promoter regions. Negative controls were either a region upstream of the *bfpA* PerA binding site [27] or the *perA* coding region (data not shown). Gels were stained with ethidium bromide and visualized with an Alpha-Imager UV transilluminator (Alpha Innotech).

### PerA CTD Structural Modeling

A computational model of the three-dimensional structure of PerA (P43459) was built with the CPHmodels 3.0 Server (http://www.cbs.dtu.dk/services/CPHmodels) [30]. After an initial search, CPHmodels defined the *E. coli* transcriptional factor Rob (PDB entry 1D5Y chain A) as the best hit to be used as a template. These two proteins share 27% of identity (data not shown). In order to refine the model, this was minimized by using the Gromacs server (http://lorentz.immstr.pasteur.fr/gromacs) [31]. Finally, the RAMPAGE program (http://mordred.bioc.cam.ac.uk/~rapper) was used to validate the stereochemical quality of the resulting three-dimensional model [32]. After analyzing the Ramachandran plot, 98.2% of the residues were located in allowed regions. All the bond distances, angles and dihedrals fulfill the normal limits for polypeptide chains. The resulting model includes 112 of the 274 residues at the carboxyl terminus of PerA. Finally, the structure was displayed in ribbon by using Pymol program.

## Results

### Mutations at the NTD Affect the Activation Function of PerA

**Figure 1:**
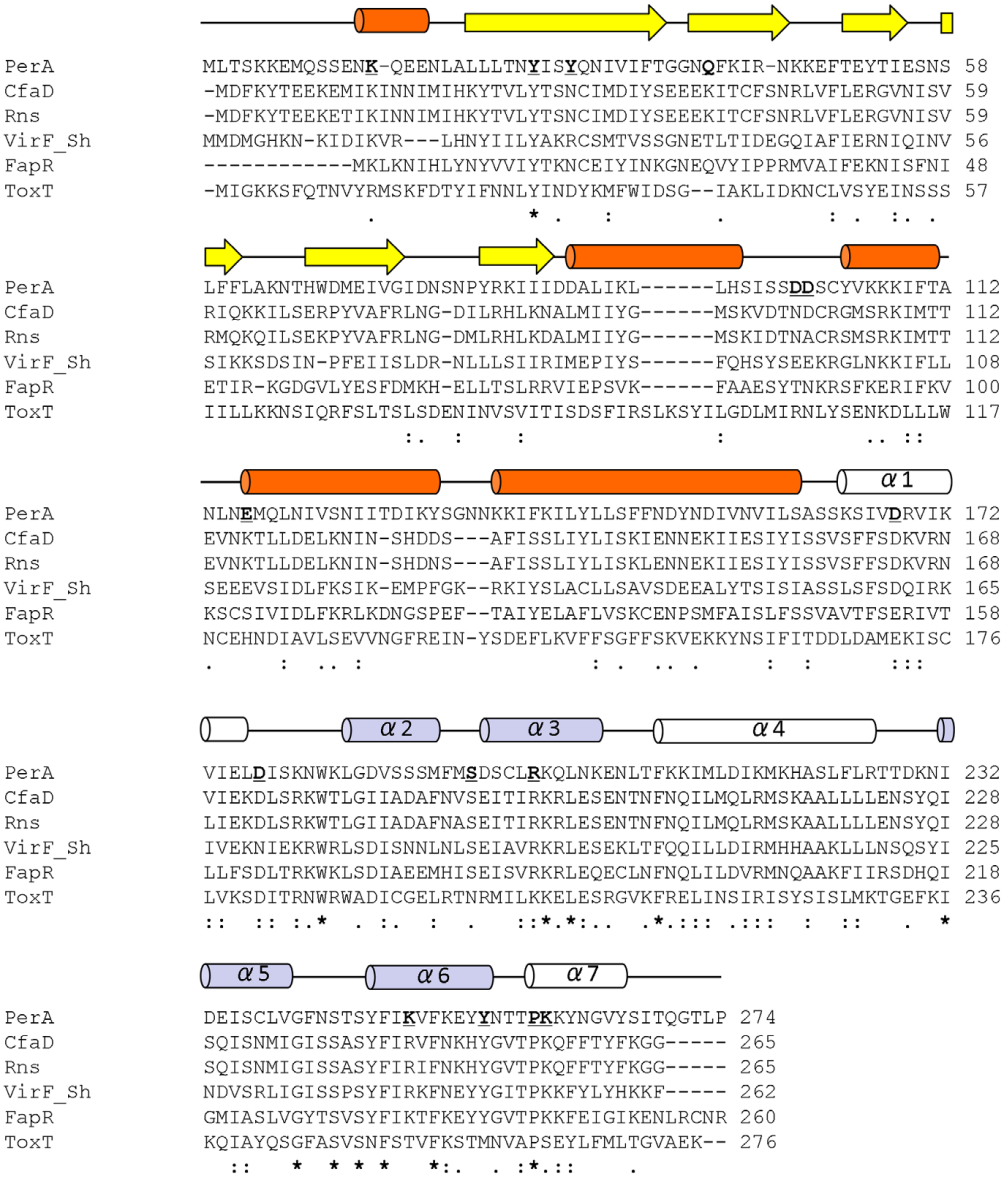
PerA residues analyzed in this study. ClustalW2 [34] alignment of PerA with evolutionary related AraC/XylS family members Rns, CfaD, VirF, FapR and ToxT. Highly conserved residues are marked by an asterisk (*), conserved residues by two dots (:) and semi-conserved by one dot (.). PerA amino acid sequence was analyzed using Psipred. α-helices are represented by orange cylinders, β-strands by yellow arrows and unstructurated regions (loops) by lines in the amino terminal domain (NTD). The predicted seven α-helices (α1–α7) at the carboxyl terminal domain (CTD) containing the predicted AraC/XylS-like HTH domain [20], are indicated above the sequences as white and blue colored cylinders. Light blue cylinders denote structures forming part of the HTH motifs in agreement with the MarA, Rob and ToxT crystallized structures [42], [44], [47]. Point mutations generated in this work are underlined and in bold.

In order to further characterize the functional role of the N-terminal domain (NTD) in PerA, we first defined the two domains in this protein by using Psipred [33]. NTD was delimited from residues 1–160 and the CTD from residues 163–274 with a possible short linker between residues 161 and 163 (Figure 1). Then, by using ClustalW2 [34], we aligned the PerA amino acid sequence with that of other virulence related AraC-like regulators such as *Shigella flexneri* VirF (VirF_Sh_), enterotoxigenic *E. coli* (ETEC) CfaD, FapR and Rns and *Vibrio cholerae* ToxT (Figure 1). With this information, amino acid residues were selected for site-directed mutagenesis depending on their location and level of conservation with the above mentioned AraC/XylS-lke regulators. Here is a brief description of those selected for this study: Residues K14 and Y26 are well conserved in all the other virulence regulators (Figure 1). Residues Y29 is not conserved but located in the same β-strand as Y26, while Q40 is also a not conserved residue located near the β1-loop-ß2 containing residue G37 that was previously shown to be required for PerA activity [24]. Residues D100, D101, and E116 are not conserved at the same positions in the other AraC-like proteins and were selected for analysis because in XylS a similar (D) residue located close to this region have been shown to interact with subunits of the RNA polymerase [35], [36]. In addition, we also wanted to learn whether elimination of segments at the NTD would affect protein function as activator. Mutants with deletions in three different segments containing putative α-helices or β-sheets in this domain were constructed (Δ30–49, Δ70–102 and Δ90–176, this latter construct also deleted the first α-helix of the CTD). Except for mutant Q40R, all changes at the NTD were alanine substitutions.

**Figure 2:**
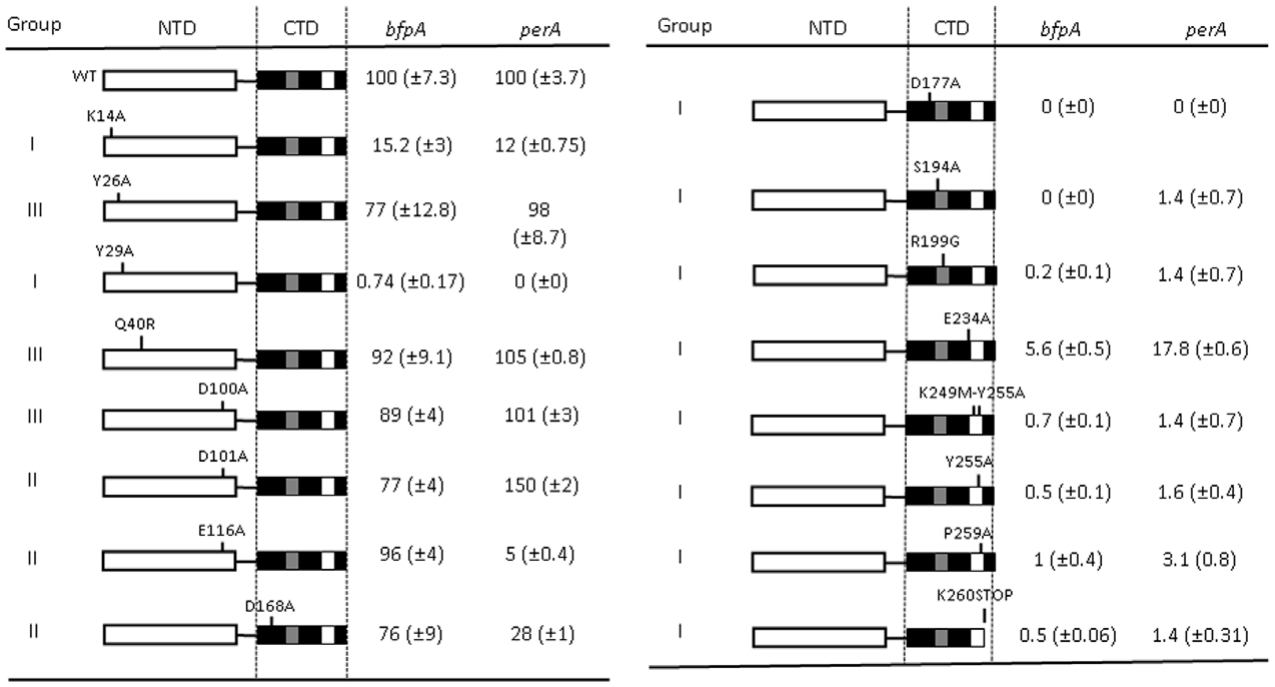
NTD and CTD mutations in PerA affect its activator function. Plasmids encoding the mutated versions of *perA* were transformed into EPEC B171-10 strain and bacteria were grown in DMEM at 37°C to OD_600_ = 1.4. One ml samples were taken to prepare cell extracts and determination of CAT activity. Data are expressed as a percentage of expression compared to that obtained with the wild type protein (wt) for each fusion. Mutants were clustered in three groups according to their phenotypes: I, severely affected (<15% of activity); II, moderately affected (>15% to <75% of activity) but able to activate only one of the fusions or that have an apposite effect on promoter activation; and III, not affected or similar to wt (>75% of activity). Values are the mean of three independent assays. Standard deviations (± SD) are shown in parenthesis.

Plasmids encoding the different *perA* variants (Table 1) were transformed into the pEAF-cured EPEC strain B171-10 to complement the expression of the *bfpA-cat* or the *perA-cat* fusions. The resulting strains were grown in DMEM at 37°C. The mutations tested had a variety of effects on PerA activity and according to their phenotype were clustered into three groups (Figure 2 and data not shown). Group I includes PerA mutants which function was drastically affected (PerA_Y29A_, PerA_Δ30–49_, PerA_ Δ70–102_ and PerA_ Δ90–176_) or that showed only residual activity for both the *bfpA* and *perA* promoters (PerA_K14A_). Group II includes PerA mutants able to activate only one of the fusions or that have an opposite effect on promoter activation. For instance the PerA_E116A_ mutant was severely affected for the activation of the *perA* promoter, but not of the *bfpA* promoter, while mutant PerA_D101A_ showed a slight reduction on its ability to activate the *bfpA* promoter, it also showed a 50% increase activation of the *perA* promoter. Group III includes variants carrying mutations with no or slight effect when compared with wild type PerA (PerA_Y26A_, PerA_Q40R_ and PerA_D100A_). Altogether, these data further demonstrate that the integrity of the NTD and specific amino acid residues are required for PerA-mediated transcriptional activation and, interestingly, provide evidence that the NTD is also important during promoter recognition and in the differential activation of both promoters.

### Both HTH Motifs in the CTD are Important for PerA Activator Function

In addition to previously identified residues in the CTD [24], here we identified additional critical residues in this domain by mutating previously uncharacterized amino acids of the first connecting loop (D177, between α1 and α2), in the two HTH motifs (HTH1 residues S194 and R199, and HTH2 residues E234, K249, Y255) and in the last α-helix (α7) P259 (Figure 1). Additionally, residue K260 was replaced by an ochre stop codon immediately after the well-conserved P259 residue generating PerA_K260STOP_, hence eliminating most of the last α-helix terminal region (α7, residues 260–274). In addition, the previously characterized residues D168, located at the first α-helix in this region, and E234, located at the HTH2 [24], were also selected for further evaluation (Figure 1).

As for the NTD mutants, the CTD variants were tested for their ability to activate the *bfp-cat* and *perA-cat* fusions in EPEC B171-10. As shown in Fig. 2, most CTD mutants at residues within or near the DNA facing helices in the HTH1 or the HTH2 motifs were unable to activate the reporter fusions (group I mutants), suggesting a defect in DNA binding, except for mutant PerA_E234A_ which was still weakly active for both promoters consistently with a previous report [24]. PerA_D168A_ mutant, located at the first CTD α-helix, showed a differential phenotype (group II mutants) being highly defective in the activation of the *perA* promoter, but only slightly diminished the activation of the *bfpA* promoter. In contrast, it was previously shown that this mutant activated a *perA-lacZ* transcriptional fusion as the wild type PerA, although was not tested for the *bfpA* promoter [24]. It is possible that the difference in results is due to the reporter gene used in each case. As for mutants PerA_D101A_ and PerA_E116A_, D168 seems to play a role in discriminating between the two promoters (see above). Finally, deletion of the last 15 amino acids (PerA_K260STOP_) also abolished the activity of the protein, suggesting that this segment plays a role either in PerA stability or in maintaining the adequate folding of the DNA binding domain (DBD). All mutants were expressed and stable as wild type PerA, as shown by western blot using an antibody directed to the last 16 amino acids of PerA [27] (Figure S1 and data not shown). PerA_K260STOP_ could not be detected because it lacks the C-terminal residues recognized by the antibody. These results further support the notion that highly conserved residues at the HTH motifs are critical for the transcriptional activation function of PerA, as for other AraC-like proteins.

#### PerA amino acid residues involved in environmental regulation

**Figure 3:**
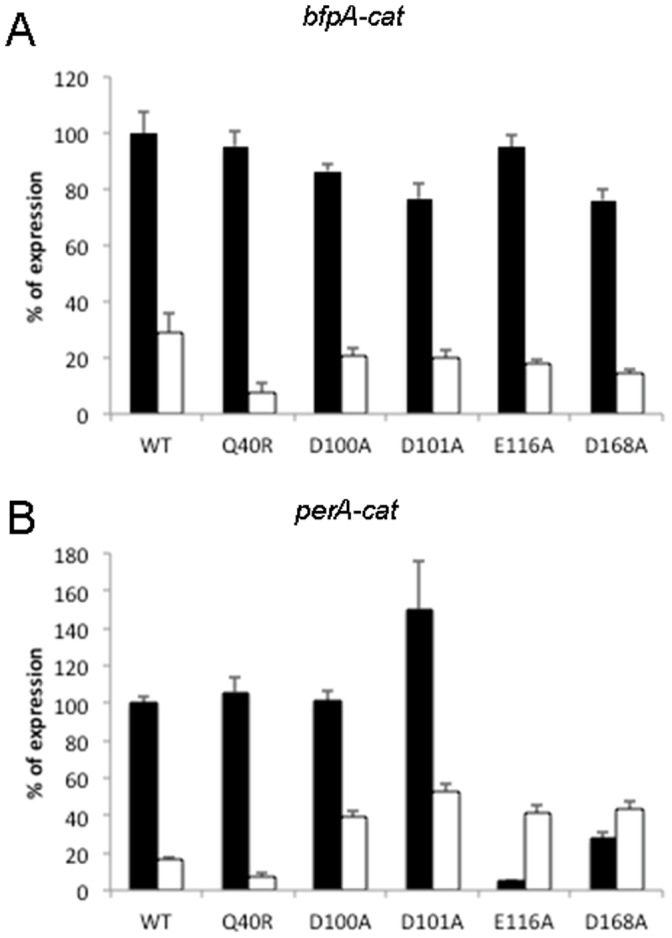
Mutations in the PerA NTD overcome or enhance ammonium repression. EPEC strain B171-10 was transformed with either *bfpA-cat* or *perA-cat* fusions and complemented with plasmids encoding either wild type (wt) or mutated proteins. Bacteria were grown in DMEM at 37°C with (white bars) or without (black bars) ammonium sulfate. CAT specific activity was determined from bacterial cultures grown to an OD_600_ of 1.0. Data are expressed as a percentage of expression compared to that obtained with the wild type protein (wt) for each fusion. The results are the mean of three independent experiments and error bars represent the standard deviation.

The expression of pEAF-encoded *bfp* and *per* genes is repressed in the presence of ammonium salts in the culture medium at 37°C [15], [22]. In order to evaluate if any of the residues analyzed in this work is involved in sensing directly or indirectly environmental signals such as ammonium salts, we determined whether group II or III PerA mutants (PerA_Y26A_, PerA_Q40R_, PerA_D100A_, PerA_D101A_, PerA_E116A_ and PerA_D168A_) were able to overcome the repressing conditions generated by ammonium salts. Despite the fact that none of the mutants was able to overcome completely the negative regulation imposed by the addition of ammonium salts some of them showed interesting phenotypes. PerA_D100A_ and PerA_D101A_ were able to slightly overcome this negative regulation but only for the activation of the *perA* promoter (Figure 3B). Notably, PerA_E116A_ showed an interesting behavior in the presence of ammonium salts, while being repressed for the activation of *bfpA* (Figure 3A), its activity was partially restored for the activation of *perA* (Fig. 3B). PerA_D168A_ was able to activate partially *perA* and this activation was insensitive to ammonium repression. As with wt PerA, activation of both promoters was still repressed in the presence of ammonium for mutants PerA_Y26A_ and PerA_Q40R_ (data not shown). These results indicate that PerA also contains residues that may play a role in sensing environmental conditions, such as the presence of ammonium salts in the medium, which have a negative effect in PerA activity. Moreover, these data further support the notion that the interaction of PerA with both promoters is different.

### Residues in both NTD and CTD have a Role in PerA DNA Binding Ability

PerA acts as an activator of the *bfp* and *per* operons by binding to their promoter regions [27] possibly by eliminating H-NS negative regulation [24]. Based on the secondary and tertiary structures of PerA (Figure 1), it is predicted that some of the defective mutants described above, in particular those containing mutations at the HTH motifs (specially those possibly facing the DNA in helices α3 and α6), would be affected in their ability to interact with DNA. We had successfully purified a functional fusion to the maltose binding protein (MBP) and used it to determine the PerA binding sites at both the *bfpA* and *perA* promoters [27]. Here we used the same strategy to test the DNA binding activity of different mutants.

**Figure 4:**
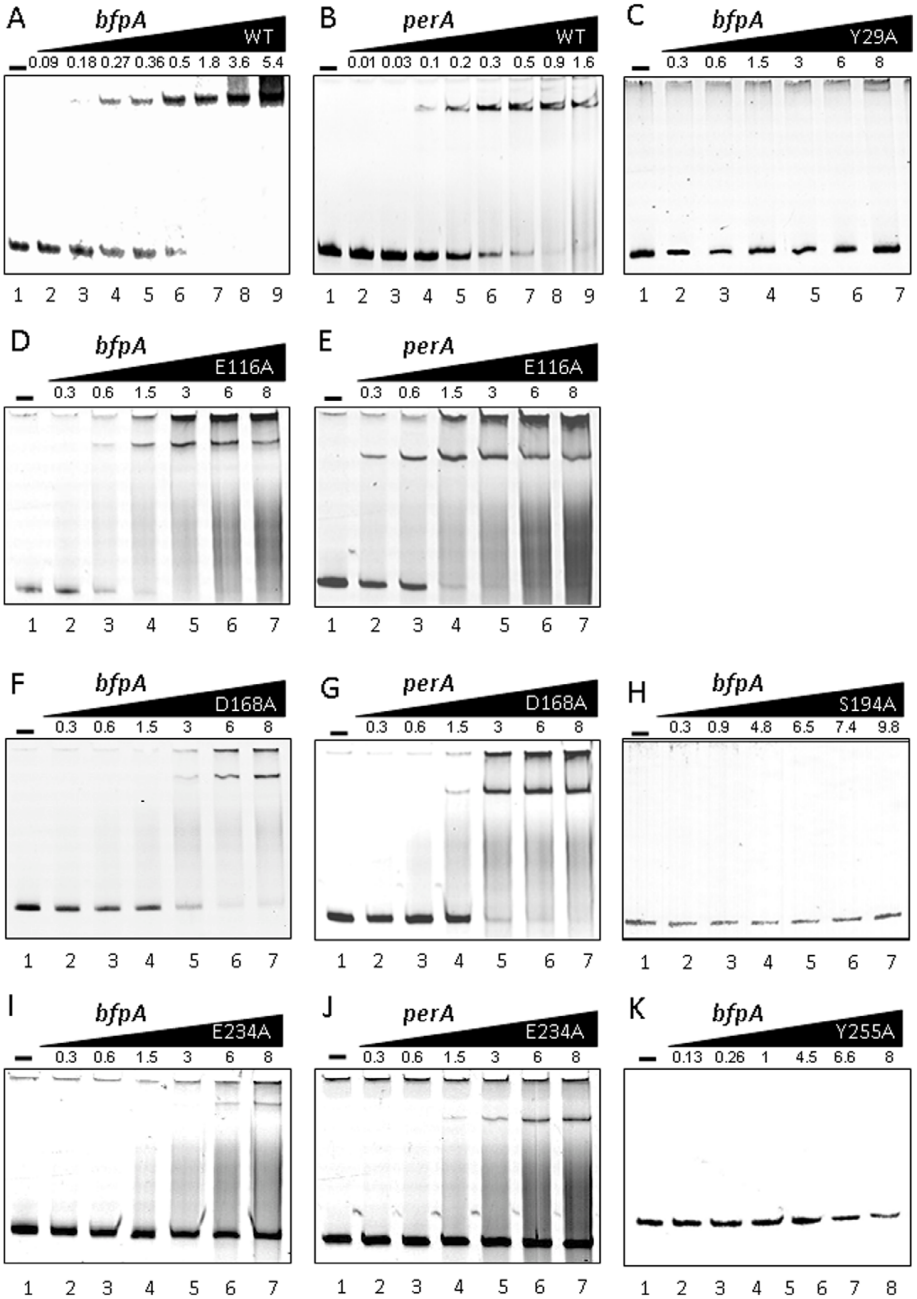
DNA binding of different PerA mutants. EMSA experiments with wild type (A and B) and mutated MBP-PerA fusions (C–K) showing the interaction with DNA fragments spanning either the *bfpA* or *perA* promoter regions (indicated above each panel). Increasing amounts in µM concentrations of each protein were used as indicated above each individual gel. Free DNA and protein-DNA complexes were resolved in 6% polyacrylamide gel electrophoresis at 120 V in 0.25X TBE gels and stained with ethidium bromide. For presentation purposes the color of all images was inverted from the original image. A similar result was obtained for the *perA* promoter region with mutants PerA_Y29A_, PerA_S194A_ and PerA_Y255A_ (data not shown).

Selected mutants were fused in-frame to the gene encoding the MBP and the resulting constructs were used to over-express them. Upon purification, their DNA binding ability was tested by electrophoretic mobility shift assays (EMSA) using increasing concentrations of the purified MBP-PerA fusion proteins and DNA fragments spanning the promoter regions of *bfpA* (positions −214 to −15 with respect to the transcriptional start site) and of *perA* (positions −155 to +21) (Figure 4). Similarly to previous results from our laboratories [27], we observed a single band shifted by MBP-PerA for both promoters, which correlates with its putative monomeric nature (Figure 4A and 4B). This contrast with the result obtained by Porter *et al.* [24] where they observed two shifted bands, it is possible that the difference is due to tag used in each case. The MBP-PerA_Y29A_ was not able to bind to any of the two promoter regions tested (Figure 4C), consistently with their observed inability to activate both transcriptional fusions (Figure 2), indicating that the NTD plays a role in DNA binding.

PerA_E116A_ mutant interacted with both promoter fragments in a similar fashion as that observed with the wt protein (Figure 4D and 4E)despite the fact that it was defective for *perA* activation, but not for the activation of *bfpA* (Figure 2). PerA_D168A_ was also able to bind to both promoters although with slightly less affinity than the wt (4F and 4G). This mutant also showed a differential activation for *bfpA* and *perA* (Figure 2). These results indicate that these residues are not involved in DNA recognition (as for E116A) or may have a slight defect in it (as for D168A) and therefore they may have other roles that affect PerA regulatory activity.

Mutations in regions HTH1 (PerA_S194A_, PerA_D177A_ and PerA_R199G_), HTH2 (PerA_K249M-Y255A_, and PerA_Y255A_) and α7 (PerA_P259A_) abolished the DNA binding ability of PerA, even when an excess of purified protein was used (Figure 4H, 4K and data not shown). PerA_E234A_ showed some interaction with both promoter regions when high concentrations of protein were used (Figure 4I and 4J), which would explain the low activation observed for both promoters (Figure 2). Altogether, these results show the importance, or the lack of it, of defined NTD and CTD PerA residues in both the NTD and CTD for DNA binding, helping us to understand the observed defects in transcriptional activation.

## Discussion

PerA plays a central role in controlling directly or indirectly the expression of several key virulence genes in EPEC [14], [16], [17], [22], [23], [24], [25], [37], including the *bfp* and *per* operons located in the pEAF (also called pB171) plasmid. Although the role of different PerA residues in gene activation has previously been reported [24], here we have expanded this analysis by assessing the role of residues not previously studied of both the NTD and CTD, in the transcriptional activation of the *perA* and *bfpA* promoters, as well as in DNA binding abilities and in the response to repression by ammonium salts.

Similar to what has been reported for Rns, VirF_Sh_ and ToxT [38], [39], [40], [41], the deletion of discrete and long NTD regions (data not shown) or alanine substitutions of single amino acids in this domain resulted in PerA mutants with impaired activation and DNA binding functions at both promoters. Similarly, NTD PerA mutants G37W, W68G and L94P were not able to activate a *perA-lacZ* transcriptional fusion [24], but the nature of this defect was not determined. In agreement with their defect *in vivo,* here we show that the purified mutants were not able to bind to the promoter DNA fragments *in vitro*. Together our results and those by others support the notion that the NTD plays a critical role in PerA activity and indirectly in DNA binding.

Interaction between both domains NTD and CTD for ToxT and other AraC-like proteins has been studied [42], [43]. For ToxT the NTD and CTD form a “closed conformation” clamped onto palmitoleic acid and makes ToxT unable to dimerize and activate transcription. Contrary, in PerA it is possible that the NTD and the CTD domains interact forming a constitutive “open conformation” allowing PerA to bind to *bfpA* and *perA* promoters. Given that no effector molecule has been found for PerA mutations at the NTD may cause a structural change that generates an inactive “close conformation”, as the one induced by fatty acids in ToxT [42], [43] or, alternatively, may induce a conformational change that also affects the conformation of the DNA binding domain. However, these and other possibilities should be further evaluated.

Interestingly, mutations in residues D101, E116 and D168, located in positions near the linker region between the NTD and CTD, rendered PerA mutants with a different activation phenotype depending on the promoter tested, PerA_D101A_ showed opposite phenotypes, while slightly less activate for the *bfpA* promoter, it showed increased activity for *perA* (around 77% versus 150%, respectively, in contrast to wild type PerA). PerA_E116A_ was able to interact with both promoter regions *in vitro* in a similar manner as the wt protein, but the mutation had a drastic negative effect in the activation of the *perA* promoter, without affecting *bfpA* activation. Moreover, PerA_D168A_ still bound to both promoters but showed about a 5- to 6- fold reduction in affinity, as estimated by EMSA (Figure 4F and 4G), which intriguingly had a more marked effect on the activation of the *perA* promoter (72% down regulation for *perA*, versus 24% for *bfpA* in comparison to wt PerA). Therefore, and given the results obtained in our study, our current model speculates that these residues differentially participate in protein-protein interactions with one or more RNAP subunits or, alternatively, that these mutations cause conformational changes in PerA that differentially influence promoter recognition and thus activation. Both hypotheses remain to be further tested.

As expected from what has been seen in other studies in AraC-like proteins, most of the mutations in the CTD domain (or DBD) abolished PerA transcriptional activation. Here we extended the observations made by Porter and collaborators [24] on the importance of the CTD by performing DNA binding assays with purified proteins.

**Figure 5:**
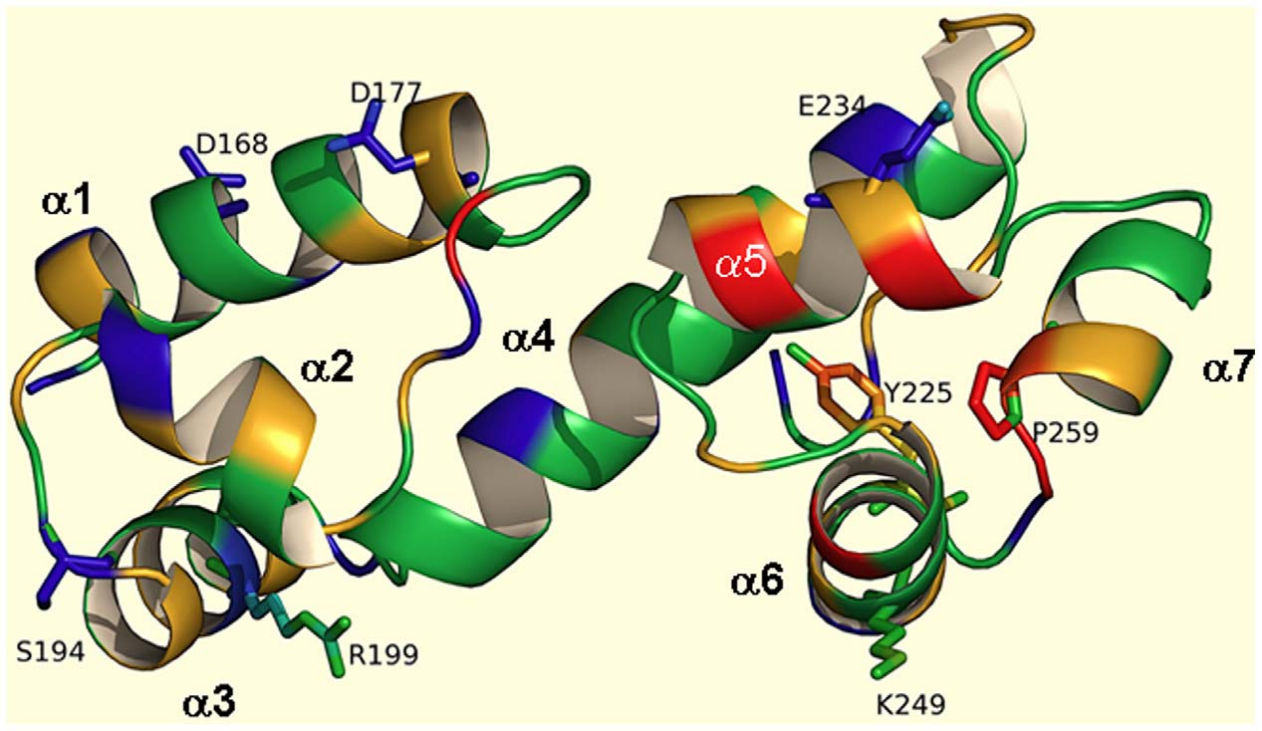
Mapping of mutations in PerA CTD. Ribbon-type model of PerA CTD predicted molecular structure, including the HTH DNA binding domain obtained with CPHmodels 3.0 (see *Materials and Methods* section) and using the Rob structure as a template. The CTD is composed of seven α-helices (α1 to α7) comprising two HTH motifs (α2-turn-α3 for HTH1 and α5-turn-α6 for HTH2) connected by a linker α-helix structure (α4) and two flanking α-helices (α1 and α7). Mutated residues D168, D177, S194, R199, Y225, E234, K249 and P259 are indicated and highlighted as sticks structures. Alpha helixes (α) correspond to those shown in Figure 1.

A three-dimensional model helped us to predict the orientation of mutated residues in the CTD (Figure 5). Despite the fact that PerA is phylogenetically more related to other virulence regulators in the AraC/XylS family [21], the CTD model is based on the Rob protein DBD because it was the best hit as a template after threading it to other crystal structures using the CPHmodels program. Given their significant sequence similarity, the conserved DBD of other AraC/XylS family proteins are likely to adopt a similar tertiary structure [20], [26]. The mutation in D177, which has a similar charge to that in MarA (D22) and SoxS (E16), was severely affected in both activation and DNA binding. An alanine substitution of the corresponding ToxT D181 residue affected the expression of the *V. cholerae* accessory colonization factor (*acf*) gene, but not that of the cholera toxin (*ctx*) gene [39]. The prediction that PerA D177 is not facing the DNA, suggests that it might be making contacts with other amino acids, perhaps with residues at the PerA NTD. Furthermore, PerA residues R199 and K249 are located in α-helices that are most likely facing the DNA, while S194 is in a connecting loop in the same orientation, supporting the idea that these residues are involved in direct interactions with either nucleotide bases or with the phosphodiester backbone. Residues Y255 and P259 are facing to the internal section of this domain suggesting they may have a role in the structural conformation adopted by the DBD. In support of this notion, Y255 is mainly conserved in AraC-like virulence regulators that are more related to PerA [21]. At this position, most of the family members contain a hydrophobic amino acid, which, according to the MarA crystal structure [44], is within the hydrophobic surface of the HTH2 motif facing hydrophobic surfaces on the other HTH subdomain, making it important for structure integrity.

PerA residue P259 is one of most conserved amino acids in the AraC/XylS family and is part of the family consensus sequence [20]. According to the MarA, ToxT and Rob structures, the equivalent proline is not exposed, as predicted for PerA in our model (Figure 5). The PerA_P259A_ mutant was severely defective for both transcription activation and DNA binding, suggesting that this residue plays an important role in structuring the DBD.

According to the structure of the MarA-*mar* co-crystal [44], [45], the first α-helix (α1) has no interaction with the DNA, though for SoxS some of the residues have been predicted to make van der Waals contacts with nucleotide bases [46]. PerA residues D168 and D177 are located in α1 (Figure 5). For D168 we observed a slight defect in *bfpA* activation but a stronger defect in *perA*. DNA shift experiments showed a slight defect in the ability of this protein to interact with both DNA fragments. It is possible that this deficiency accounts for the expression observed in *bfpA* but does not explain the even more drastic defect in *perA* (Figure 2). We hypothesize that this residue is involved in correct folding of the DBD that yields a defected CTD. Alternatively it is possible that D168 is involved also in differential interactions with the RNAP.

We have shown that gene regulation in EPEC is particularly sensitive to the presence of ammonium ions, which represses the expression of both operons [15], [22]. Interestingly, PerA mutant Q40R, which is active as the wt protein in DMEM, further repressed the expression of both fusions in the presence of ammonium. Although the molecular mechanism underlying ammonium repression remains unknown, this result suggested that this residue might be part of a functional motif involved in the interaction with a putative EPEC specific negative regulatory molecule that is produced or activated in the presence of ammonium ions. We cannot conclusively rule out the possibility that the ammonium ions are directly exerting this negative effect, but the fact that both promoter fusions are expressed in a non-EPEC background (*e.g. E. coli* K12), when complemented with wt PerA in the presence of ammonium, argues against this possibility [22]. Furthermore, we also observed that mutants PerA_E116A_ and PerA_D168A_ were less sensitive to ammonium repression, but only when activating the *perA* promoter, suggesting that albeit having a dramatic effect on *perA* expression, these mutations also generated a conformational change that partially suppressed this defect.

In summary, this study expands the knowledge regarding the critical role that both the NTD and CTD play in DNA binding and PerA-mediated transcriptional activation of the *per* and *bfpA* promoters. It also provides evidence that PerA differentially interacts with both promoters and may possess a putative functional motif that makes it susceptible to ammonium inhibition.

## Supporting Information

Figure S1 **Expression of PerA variants.** Different PerA mutants were expressed in EPEC strain B171-10 and detected by Western blot using anti-PerA antibodies. Whole cell extracts were resolved in a 10% SDS-PAGE, transferred to a nitrocellulose membrane, blocked with 5% non-fat milk and blotted with 1∶2000 anti-PerA antibodies. B171-10 carrying the empty vector was used as a negative control (Neg. First lane) and purified MBP-PerA was used as a positive control (last lane). A smaller unspecific band (indicated with an asterisk) worked as a loading control. Indicated are the respective mutated residues for each PerA variant. The arrow shows the band corresponding to PerA.
(PPTX)

## References

[1] Chen HD, Frankel G (2005) Enteropathogenic *Escherichia coli*: unravelling pathogenesis. FEMS Microbiol Rev 29: 83–98. 15652977 10.1016/j.femsre.2004.07.002

[2] Nataro JP, Kaper JB (1998) Diarrheagenic *Escherichia coli* . Clinical Microbiol Rev 11: 142–201. 9457432 10.1128/cmr.11.1.142 PMC121379

[3] Kaper JB, Nataro JP, Mobley HL (2004) Pathogenic *Escherichia coli* . Nat Rev Microbiol 2: 123–140. 15040260 10.1038/nrmicro818

[4] Humphries RM, Armstrong GD (2010) Sticky situation: localized adherence of enteropathogenic *Escherichia coli* to the small intestine epithelium. Future Microbiol 5: 1645–1661. 21133687 10.2217/fmb.10.124

[5] Elliott SJ, Sperandio V, Giron JA, Shin S, Mellies JL, et al (2000) The locus of enterocyte effacement (LEE)-encoded regulator controls expression of both LEE- and non-LEE-encoded virulence factors in enteropathogenic and enterohemorrhagic *Escherichia coli* . Infect Immun 68: 6115–6126. 11035714 10.1128/iai.68.11.6115-6126.2000 PMC97688

[6] Campellone KG, Leong JM (2003) Tails of two Tirs: actin pedestal formation by enteropathogenic *E. coli* and enterohemorrhagic *E. coli* O157: H7. Curr Op Microbiol 6: 82–90. 10.1016/s1369-5274(03)00005-5 12615225

[7] Giron JA, Ho AS (1991) Schoolnik GK (1991) An inducible bundle-forming pilus of enteropathogenic *Escherichia coli* . Science 254: 710–713. 1683004 10.1126/science.1683004

[8] Bieber D, Ramer SW, Wu CY, Murray WJ, Tobe T, et al (1998) Type IV pili, transient bacterial aggregates, and virulence of enteropathogenic *Escherichia coli* . Science 280: 2114–2118. 9641917 10.1126/science.280.5372.2114

[9] Cleary J, Lai LC, Shaw RK, Straatman-Iwanowska A, Donnenberg MS, et al (2004) Enteropathogenic *Escherichia coli* (EPEC) adhesion to intestinal epithelial cells: role of bundle-forming pili (BFP), EspA filaments and intimin. Microbiology 150: 527–538. 14993302 10.1099/mic.0.26740-0

[10] Sohel I, Puente JL, Ramer SW, Bieber D, Wu CY, et al (1996) Enteropathogenic *Escherichia coli*: identification of a gene cluster coding for bundle-forming pilus morphogenesis. J Bacteriol 178: 2613–2628. 8626330 10.1128/jb.178.9.2613-2628.1996 PMC177987

[11] Stone KD, Zhang HZ, Carlson LK, Donnenberg MS (1996) A cluster of fourteen genes from enteropathogenic *Escherichia coli* is sufficient for the biogenesis of a type IV pilus. Mol Microbiol 20: 325–337. 8733231 10.1111/j.1365-2958.1996.tb02620.x

[12] Sohel I, Puente JL, Murray WJ, Vuopio-Varkila J (1993) Schoolnik GK (1993) Cloning and characterization of the bundle-forming pilin gene of enteropathogenic *Escherichia coli* and its distribution in *Salmonella* serotypes. Mol Microbiol 7: 563–575. 8096320 10.1111/j.1365-2958.1993.tb01147.x

[13] Donnenberg MS, Giron JA, Nataro JP, Kaper JB (1992) A plasmid-encoded type IV fimbrial gene of enteropathogenic *Escherichia coli* associated with localized adherence. Mol Microbiol 6: 3427–3437. 1362446 10.1111/j.1365-2958.1992.tb02210.x

[14] Bustamante VH, Calva E, Puente JL (1998) Analysis of cis-acting elements required for bfpA expression in enteropathogenic *Escherichia coli* . J Bacteriol 180: 3013–3016. 9603898 10.1128/jb.180.11.3013-3016.1998 PMC107275

[15] Puente JL, Bieber D, Ramer SW, Murray W (1996) Schoolnik GK (1996) The bundle-forming pili of enteropathogenic *Escherichia coli*: transcriptional regulation by environmental signals. Mol Microbiol 20: 87–100. 8861207 10.1111/j.1365-2958.1996.tb02491.x

[16] Gomez-Duarte OG, Kaper JB (1995) A plasmid-encoded regulatory region activates chromosomal *eaeA* expression in enteropathogenic *Escherichia coli* . Infect Immun 63: 1767–1776. 7729884 10.1128/iai.63.5.1767-1776.1995 PMC173222

[17] Tobe T (1996) Schoolnik GK, Sohel I, Bustamante VH, Puente JL (1996) Cloning and characterization of *bfpTVW*, genes required for the transcriptional activation of *bfpA* in enteropathogenic *Escherichia coli* . Mol Microbiol 21: 963–975. 8885267 10.1046/j.1365-2958.1996.531415.x

[18] Iida M, Okamura N, Yamazaki M, Yatsuyanagi J, Kurazono T, et al (2010) Classification of *perA* sequences and their correlation with autoaggregation in typical enteropathogenic *Escherichia coli* isolates collected in Japan and Thailand. Microbiol Immunol 54: 184–195. 20377747 10.1111/j.1348-0421.2010.00212.x

[19] Okeke IN, Borneman JA, Shin S, Mellies JL, Quinn LE, et al (2001) Comparative sequence analysis of the plasmid-encoded regulator of enteropathogenic *Escherichia coli* Strains. Infect Immun 69: 5553–5564. 11500429 10.1128/IAI.69.9.5553-5564.2001 PMC98669

[20] Gallegos MT, Schleif R, Bairoch A, Hofmann K, Ramos JL (1997) Arac/XylS family of transcriptional regulators. Microbiol Mol Biol Rev 61: 393–410. 9409145 10.1128/mmbr.61.4.393-410.1997 PMC232617

[21] Ibarra JA, Perez-Rueda E, Segovia L, Puente JL (2008) The DNA-binding domain as a functional indicator: the case of the AraC/XylS family of transcription factors. Genetica 133: 65–76. 17712603 10.1007/s10709-007-9185-y

[22] Martinez-Laguna Y, Calva E, Puente JL (1999) Autoactivation and environmental regulation of *bfpT* expression, the gene coding for the transcriptional activator of *bfpA* in enteropathogenic *Escherichia coli* . Mol Microbiol 33: 153–166. 10411732 10.1046/j.1365-2958.1999.01460.x

[23] Mellies JL, Elliott SJ, Sperandio V, Donnenberg MS, Kaper JB (1999) The Per regulon of enteropathogenic *Escherichia coli*: identification of a regulatory cascade and a novel transcriptional activator, the locus of enterocyte effacement (LEE)-encoded regulator (Ler). Mol Microbiol 33: 296–306. 10411746 10.1046/j.1365-2958.1999.01473.x

[24] Porter ME, Mitchell P, Roe AJ, Free A, Smith DG, et al (2004) Direct and indirect transcriptional activation of virulence genes by an AraC-like protein, PerA from enteropathogenic *Escherichia coli* . Mol Microbiol 54: 1117–1133. 15522091 10.1111/j.1365-2958.2004.04333.x

[25] Bustamante VH, Villalba MI, Garcia-Angulo VA, Vazquez A, Martinez LC, et al (2011) PerC and GrlA independently regulate Ler expression in enteropathogenic *Escherichia coli* . Mol Microbiol 82: 398–415. 21895790 10.1111/j.1365-2958.2011.07819.x

[26] Egan SM (2002) Growing repertoire of AraC/XylS activators. J Bacteriol 184: 5529–5532. 12270809 10.1128/JB.184.20.5529-5532.2002 PMC139625

[27] Ibarra JA, Villalba MI, Puente JL (2003) Identification of the DNA binding sites of PerA, the transcriptional activator of the *bfp* and *per* operons in enteropathogenic *Escherichia coli* . J Bacteriol 185: 2835–2847. 12700263 10.1128/JB.185.9.2835-2847.2003 PMC154397

[28] Sambrook J, Russell WR (2001) Molecular cloning: a laboratory manual; Sambrook J, editor. Cold Spring Harbor, NY: CSHL Press.

[29] Meza R, Nunez-Valdez ME, Sanchez J, Bravo A (1996) Isolation of Cry1Ab protein mutants of *Bacillus thuringiensis* by a highly efficient PCR site-directed mutagenesis system. FEMS Microbiol Lett 145: 333–339. 8978087 10.1111/j.1574-6968.1996.tb08597.x

[30] Nielsen M, Lundegaard C, Lund O, Petersen TN (2010) CPHmodels-3.0–remote homology modeling using structure-guided sequence profiles. Nucleic Acids Res 38: W576–581. 20542909 10.1093/nar/gkq535 PMC2896139

[31] Lindahl E, Azuara C, Koehl P, Delarue M (2006) NOMAD-Ref: visualization, deformation and refinement of macromolecular structures based on all-atom normal mode analysis. Nucleic Acids Res 34: W52–56. 16845062 10.1093/nar/gkl082 PMC1538881

[32] Furnham N, Dore AS, Chirgadze DY, de Bakker PI, Depristo MA, et al (2006) Knowledge-based real-space explorations for low-resolution structure determination. Structure 14: 1313–1320. 16905105 10.1016/j.str.2006.06.014

[33] Buchan DW, Ward SM, Lobley AE, Nugent TC, Bryson K, et al (2010) Protein annotation and modelling servers at University College London. Nucleic Acids Res 38: W563–568. 20507913 10.1093/nar/gkq427 PMC2896093

[34] Larkin MA, Blackshields G, Brown NP, Chenna R, McGettigan PA, et al (2007) Clustal W and Clustal X version 2.0. Bioinformatics 23: 2947–2948. 17846036 10.1093/bioinformatics/btm404

[35] Ruiz R, Ramos JL (2002) Residues 137 and 153 at the N terminus of the XylS protein influence the effector profile of this transcriptional regulator and the sigma factor used by RNA polymerase to stimulate transcription from its cognate promoter. J Biol Chem 277: 7282–7286. 11751934 10.1074/jbc.M110226200

[36] Ruiz R, Ramos JL (2001) Residues 137 and 153 of XylS influence contacts with the C-terminal domain of the RNA polymerase alpha subunit. Biochem Biophysic Res Comm 287: 519–521. 10.1006/bbrc.2001.5615 11554759

[37] Tobe T, Tatsuno I, Katayama E, Wu CY (1999) Schoolnik GK, et al (1999) A novel chromosomal locus of enteropathogenic *Escherichia coli* (EPEC), which encodes a *bfpT*-regulated chaperone-like protein, TrcA, involved in microcolony formation by EPEC. Mol Microbiol 33: 741–752. 10447884 10.1046/j.1365-2958.1999.01522.x

[38] Basturea GN, Bodero MD, Moreno ME, Munson GP (2008) Residues near the amino terminus of Rns are essential for positive autoregulation and DNA binding. J Bacteriol 190: 2279–2285. 18223083 10.1128/JB.01705-07 PMC2293220

[39] Childers BM, Weber GG, Prouty MG, Castaneda MM, Peng F, et al (2007) Identification of residues critical for the function of the *Vibrio cholerae* virulence regulator ToxT by scanning alanine mutagenesis. J Mol Biol 367: 1413–1430. 17320105 10.1016/j.jmb.2007.01.061

[40] Mahon V, Smyth CJ, Smith SG (2010) Mutagenesis of the Rns regulator of enterotoxigenic *Escherichia coli* reveals roles for a linker sequence and two helix-turn-helix motifs. Microbiology 156: 2796–2806. 20507887 10.1099/mic.0.038521-0

[41] Porter ME, Dorman CJ (2002) In vivo DNA-binding and oligomerization properties of the *Shigella flexneri* AraC-like transcriptional regulator VirF as identified by random and site-specific mutagenesis. J Bacteriol 184: 531–539. 11751832 10.1128/JB.184.2.531-539.2002 PMC139584

[42] Lowden MJ, Skorupski K, Pellegrini M, Chiorazzo MG, Taylor RK, et al (2010) Structure of *Vibrio cholerae* ToxT reveals a mechanism for fatty acid regulation of virulence genes. Proc Nat Acad Sci USA 107: 2860–2865. 20133655 10.1073/pnas.0915021107 PMC2840316

[43] Childers BM, Cao X, Weber GG, Demeler B, Hart PJ, et al (2011) N-terminal residues of the *Vibrio cholerae* virulence regulatory protein ToxT involved in dimerization and modulation by fatty acids. J Biol Chem 286: 28644–28655. 21673111 10.1074/jbc.M111.258780 PMC3151105

[44] Rhee S, Martin RG, Rosner JL, Davies DR (1998) A novel DNA-binding motif in MarA: the first structure for an AraC family transcriptional activator. Proc Nat Acad Sci USA 95: 10413–10418. 9724717 10.1073/pnas.95.18.10413 PMC27908

[45] Gillette WK, Martin RG, Rosner JL (2000) Probing the *Escherichia coli* transcriptional activator MarA using alanine-scanning mutagenesis: residues important for DNA binding and activation. J Mol Biol 299: 1245–1255. 10873449 10.1006/jmbi.2000.3827

[46] Griffith KL, Wolf RE Jr (2002) A comprehensive alanine scanning mutagenesis of the *Escherichia coli* transcriptional activator SoxS: identifying amino acids important for DNA binding and transcription activation. J Mol Biol 322: 237–257. 12217688 10.1016/s0022-2836(02)00782-9

[47] Kwon HJ, Bennik MH, Demple B, Ellenberger T (2000) Crystal structure of the *Escherichia coli* Rob transcription factor in complex with DNA. Nature Struct Biol 7: 424–430. 10802742 10.1038/75213

