# Supplementary figures and images for: Further Characterization of Functional Domains of PerA, Role of Amino and Carboxy Terminal Domains in DNA Binding
Source: PLoS One. 2013 Feb 25;8(2):e56977. doi: 10.1371/journal.pone.0056977 (PMC3581565; doi:10.1371/journal.pone.0056977)

## Slide 1
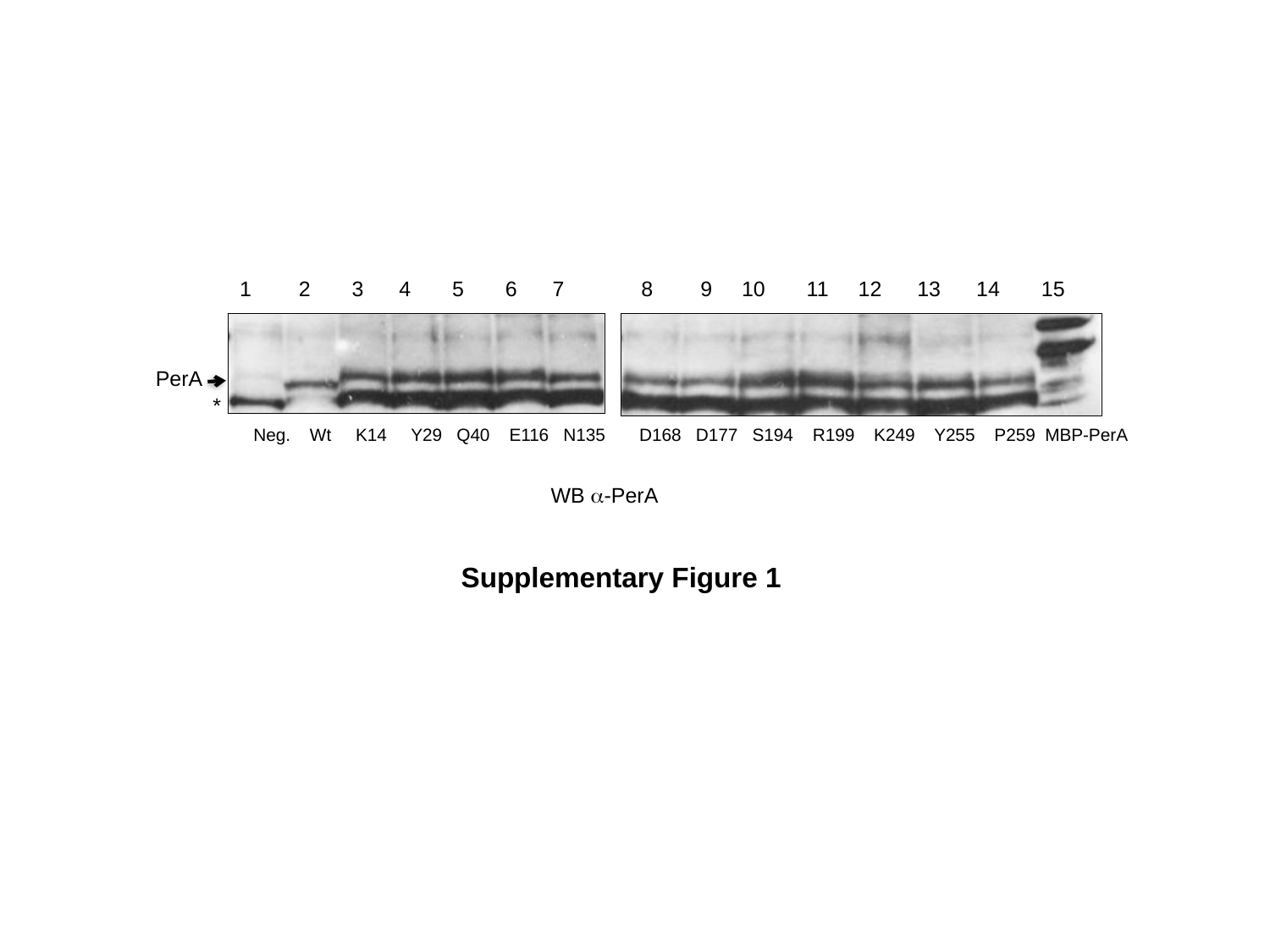

1 2 3 4 5 6 7 8 9 10 11 12 13 14 15
PerA
*
 Neg. Wt K14 Y29 Q40 E116 N135 D168 D177 S194 R199 K249 Y255 P259 MBP-PerA
WB a-PerA
Supplementary Figure 1

Supplement: Figure S1 — Expression of PerA variants. Different PerA mutants were expressed in EPEC strain B171-10 and detected by Western blot using anti-PerA antibodies. Whole cell extracts were resolved in a 10% SDS-PAGE, transferred to a nitrocellulose membrane, blocked with 5% non-fat milk and blotted with 1∶2000 anti-PerA antibodies. B171-10 carrying the empty vector was used as a negative control (Neg. First lane) and purified MBP-PerA was used as a positive control (last lane). A smaller unspecific band (indicated with an asterisk) worked as a loading control. Indicated are the respective mutated residues for each PerA variant. The arrow shows the band corresponding to PerA. (PPTX) [file pone.0056977.s001.pptx]
